# Editorial: From Residential Care to Supported Housing

**DOI:** 10.3389/fpsyt.2020.00560

**Published:** 2020-06-12

**Authors:** Angelo Barbato, Barbara D’Avanzo, Carol Harvey, Alain Lesage, Antonio Maone

**Affiliations:** ^1^Unit for Quality of Care and Rights Promotion in Mental Health, Department of Neuroscience, IRCCS Istituto di Ricerche Farmacologiche “Mario Negri”, Milano, Italy; ^2^Psychosocial Research Centre, Department of Psychiatry, University of Melbourne and NorthWestern Mental Health, Melbourne, VIC, Australia; ^3^Institut Universitaire en Santé Mentale de Montréal, CIUSSS est-de-l'île-de-Montréal, Montréal, QC, Canada; ^4^Psychiatric Residential Care Unit, Dipartimento di Salute Mentale, ASL Roma 1, Roma, Italy

**Keywords:** mental health services, community care, psychosocial rehabilitation, community residential facilities, supported housing

Residential care and supported housing are two models of accommodation for people with mental disorders in post-institutional mental health systems. In residential care, the emphasis is on treatment and rehabilitation provided by professionals in staffed facilities belonging to community psychiatric services, whereas in supported housing the emphasis is on outreach need-led support to people living on a permanent basis in their own home integrated in the community.

The supported housing approach grew from a dissatisfaction with the original model of residential facilities, developed in the early wave of downsizing or closure of mental hospitals, based on the concept of a “linear continuum”, in which persons were supposed to gradually progress from hospitals, through less supervised accommodations, halfway houses, group homes, to reach finally independent housing. However, this model failed in most cases to move people toward independent lives and trapped many people in small segregated residential settings. This was also due to the confusion between accommodation and care. Instead, a core aspect of the supported housing model is the separation between accommodation and treatment services.

The papers by Farkas and Coe and by Dorvil and Tousignant-Groulx present conceptual and historical overviews of these developments in the USA and Canada, suggesting a number of relevant questions, addressed by other papers representing the multifaceted nature of community-based residential settings. The challenges to be met include the balance of isolation versus treatment and support (Fossey et al.; Dorvil and Tousignant-Groulx), the difficulties of assessing the effectiveness of supported housing models (Killaspy et al.), and the evidence that the recovery orientation of a residential facility is not linked to facility type (Rapisarda et al.; Fletcher et al.). All papers, taken together, point out that a new home represents a turning point for people with mental health problems. Arguably, access to adequate housing is both a human right and a necessary prerequisite for recovery.

Worldwide surveys of mental health services and literature in the field both reveal an amazing array of residential solutions and a lack of agreement on the definition and classification of residential models (1). Although some recent proposals tried to lay the foundations for a coherent classification (2), this issue is still far from settled and is a barrier to practice, policy and research.

Apostolopoulou et al. and Parker et al. shed light on this by describing the characteristics of transitional residential rehabilitation models and their residents, in Greece and Australia, respectively. Fletcher et al. describe another Australian residential model focused on providing sub-acute clinical mental health care integrated with intensive recovery-focused psychosocial input.

In regard to the suitability of supported housing for all patients, independently from the degree of their autonomy, it is worth noting that although almost all participants in the “At Home” supported housing intervention experienced neurocognitive deficits, these did not prevent the achievement of housing stability (Stergiopoulos et al.), showing that housing stability can be achieved even for those who cannot be completely independent. A promising methodology to study the attributes of these diverse housing settings and associated outcomes for groups of individuals is reported by Felx et al., who developed a conceptual model of housing and community-based residential settings based on stakeholder perceptions and values, and the need to combine not always concordant views, as shown by Rapisarda et al.

Getting a house may not be all and requires, in many cases, support to get the best from living independently. This indicates that the model of supported housing should be sustained by specific and more cogent research of how support should be provided, as suggested by Fossey et al., even when involving peer workers. However, problems associated with supported housing should be acknowledged and may include housing affordability, location in unattractive neighborhoods, complex organization of outreach services, failure to provide flexible support when needed, boundary problems between health and social services, isolation of people, and safety of residents.

Clearly, closing large hospitals, questioning custodial care models, promoting supported housing, distinguishing housing from treatment, and enhancing the presence and roles of peer support workers (Fossey et al.; Rapisarda et al.; Meurk et al.) are being pursued. The latest developments will depend on integration between the social sector (housing) and the health sector (mental health care) in collaboration with policy at local and countrywide level.

Research methods like randomized trials are rarer in the social sector than the health sector, probably due to the long divide between those sectors in terms of models, financing, and power (3). Killaspy et al. present the problems in studying the efficacy of supported housing models in their unsuccessful feasibility study, pointing out consumer and staff barriers to randomization in this housing issue.

A new generation of social scientists demonstrated that policy decisions can be informed by pragmatic randomized trials of socio-political interventions: the “At Home” demonstration project in Canada (Stergiopoulos et al.) showed how a cluster randomized trial can be conducted on issues of housing stability for mentally ill homeless, with mixed methods to describe outcomes in housing and experience of improved quality of life. This confirms that the primary outcome of supported housing should be to keep people in independent accommodation, not improvement of symptoms or skills.

Attention should be paid to the risk of domination by one supported housing model. Hospital acute beds are required as well as an array of residential services in a balanced mental health system. At a given point, shelters that represent veritable social lifeboats, tertiary care facilities, supervised residential settings, or apartments may represent the best balance between the need for socialization, treatment, crisis support, rehabilitation, and autonomy. In fact, the adoption of a supported housing approach does not necessarily mean that time-limited residential alternatives to hospital admission should not be available. Nonetheless, the availability of a variety of solutions should not open the door to an uncontrolled increase of small institutions, which in turn may hinder a recovery oriented approach.

Research should prioritize evaluation of the quality of the existing residential services, standards and population-based needs, as well as more pragmatic and innovative randomized trials. The role of peer support workers in housing and home care teams should be studied with trials using mixed methods (4). Anyway, the choice of the best methods depends on the nature of the investigated issues. We should also identify meaningful questions helping mental health care to overcome custodial approaches, particularly in the area of residential and accommodation needs which is highly exposed to such risk. The paper by Farkas and Coe contains a serious warning: while evidence has been accumulating about the benefits of the supported housing model, the risk of going back to a more institutional approach, deeply present in the mental health care system, cannot be overlooked.

## Author Contributions

AB, BD'A, CH, AL, and AM contributed to the design, review, and editing of the Research Topic and to the editorial summarizing it.

## Conflict of Interest

The authors declare that the research was conducted in the absence of any commercial or financial relationships that could be construed as a potential conflict of interest.

## References

[1] Barbato A Civenti G D'Avanzo B Community residential facilities in mental health services: A ten-year comparison in Lombardy. Health Policy (2017) 121(6):623–8. 10.1016/j.healthpol.2017.03.012 28400127

[2] McPherson P Krotofil J Killaspy H What works? Toward a new classification system for mental health supported accommodation services: the simple taxonomy for supported accommodation (STAX-SA). Int J Environ Res Public Health (2018) 15(2):190. 10.3390/ijerph15020190 PMC5858263 29364171

[3] Leutz WN Five laws for integrating medical and social services: lessons from the United States and the United Kingdom. Milbank Q (1999) 77(1):77–110. 10.1111/1468-0009.00125 10197028 PMC2751110

[4] Johnson S Lamb D Marston L Osborn D Mason O Henderson C Peer-supported self-management for people discharged from a mental health crisis team: a randomised controlled trial. Lancet (2018) 392(10145):409–18. 10.1016/S0140-6736(18)31470-3 PMC6083437 30102174

